# Relation of DNA Methylation of 5′-CpG Island of *ACSL3* to Transplacental Exposure to Airborne Polycyclic Aromatic Hydrocarbons and Childhood Asthma

**DOI:** 10.1371/journal.pone.0004488

**Published:** 2009-02-16

**Authors:** Frederica Perera, Wan-yee Tang, Julie Herbstman, Deliang Tang, Linda Levin, Rachel Miller, Shuk-mei Ho

**Affiliations:** 1 The Columbia Center for Children's Environmental Health, Columbia University Mailman School of Public Health, New York, New York, United States of America; 2 Department of Environmental Health, University of Cincinnati, Cincinnati, Ohio, United States of America; 3 Center for Environmental Genetics, University of Cincinnati, Cincinnati, Ohio, United States of America; 4 Cancer Center, College of Medicine, University of Cincinnati, Cincinnati, Ohio, United States of America; 5 Division of Pulmonary, Allergy and Critical Care Medicine, Columbia University College of Physicians and Surgeons, New York, New York, United States of America; Victor Chang Cardiac Research Institute, Australia

## Abstract

In a longitudinal cohort of ∼700 children in New York City, the prevalence of asthma (>25%) is among the highest in the US. This high risk may in part be caused by transplacental exposure to traffic-related polycyclic aromatic hydrocarbons (PAHs) but biomarkers informative of PAH-asthma relationships is lacking. We here hypothesized that epigenetic marks associated with transplacental PAH exposure and/or childhood asthma risk could be identified in fetal tissues. Mothers completed personal prenatal air monitoring for PAH exposure determination. Methylation sensitive restriction fingerprinting was used to analyze umbilical cord white blood cell (UCWBC) DNA of 20 cohort children. Over 30 DNA sequences were identified whose methylation status was dependent on the level of maternal PAH exposure. Six sequences were found to be homologous to known genes having one or more 5′-CpG island(s) (5′-CGI). Of these, *acyl-CoA synthetase long-chain family member 3* (*ACSL3*) exhibited the highest concordance between the extent of methylation of its 5′-CGI in UCWBCs and the level of gene expression in matched fetal placental tissues in the initial 20 cohort children. *ACSL3* was therefore chosen for further investigation in a larger sample of 56 cohort children. Methylation of the *ACSL3* 5′-CGI was found to be significantly associated with maternal airborne PAH exposure exceeding 2.41 ng/m^3^ (OR = 13.8; *p*<0.001; sensitivity = 75%; specificity = 82%) and with a parental report of asthma symptoms in children prior to age 5 (OR = 3.9; *p*<0.05). Thus, if validated, methylated *ACSL3* 5′CGI in UCWBC DNA may be a surrogate endpoint for transplacental PAH exposure and/or a potential biomarker for environmentally-related asthma. This exploratory report provides a new blueprint for the discovery of epigenetic biomarkers relevant to other exposure assessments and/or investigations of exposure-disease relationships in birth cohorts. The results support the emerging theory of early origins of later life disease development.

## Introduction

Asthma is the most common chronic childhood disease [1] and its risk may be strongly influenced by prenatal events [2]. Low income communities experience some of the highest childhood asthma rates in the U.S [3] and new preventive strategies are lacking due in part to the absence of predictive biomarkers. In our longitudinal cohort study of children residing in urban low-income, minority communities of New York City [4]–[6] the asthma rate exceeds 25% and is among the highest in the nation. Preliminary evidence suggests that transplacental exposure to polycyclic aromatic hydrocarbons (PAHs) derived largely from traffic-related air pollutants may be a risk factor for the early development of asthma-related symptoms in this cohort [5]. However, we could not rule out the possibility that postnatal exposure of children in this cohort to these pollutants may also contribute to the symptoms without additional studies.

According to the hypothesis of the early origins of chronic disease, exposure to agents that are transmissible from mother to fetus has profound influences on the functional capacity and performance of many organs/tissues later in life [7]–[11]. Developmental plasticity allows the fetus to make anticipatory responses to the external environment by altering the course of cellular and organ differentiation *in utero* in order to gain adaptive advantages for later life challenges. However, a pronounced mismatch between “anticipatory adaptations” made during early life and demands in later life could be a cause of disease [10]. More importantly, environmental insults could “mislead” early organogenesis resulting in serious ailments in later life. The best studied mechanisms underpinning the latter scenario is the induction of aberrant DNA methylation of regulatory sequences in susceptible genes by environmental toxicants during critical developmental periods, leading to inappropriate gene expression and disease pathogenesis in later life [12]–[16]. This body of research suggests that transplacental exposure to high levels of airborne PAHs could cause aberrant DNA methylation changes, leading to dysregulation of gene expression and perhaps childhood asthma. These alterations could occur, not only in target organs/tissues such as fetal lung or the immune system [11], but also in peripheral tissues such as fetal circulating white blood cells and placental tissues, thus providing opportunities to discover and develop highly sensitive and specific, minimally invasive biomarkers for exposure assessments or clinical indications [17].

The primary objective of this study was to explore whether transplacental exposure to PAHs in humans induces epigenetic reprogramming involving aberrant DNA methylation of specific genes that might be mechanistically related to childhood asthma or airway inflammation. Study subjects were children born to nonsmoking Dominican and African American women living in Northern Manhattan and the South Bronx. They were participants in the Columbia Center for Children's Environmental Health (CCCEH) cohort study previously described [4]–[6]. Mothers completed prenatal air monitoring and donated a maternal and/or umbilical cord blood sample and, in some cases, fetal placental tissue (FPT) at delivery. Umbilical cord white blood cells (UCWBC) and matched FPT from 20 cohort subjects were used initially for candidate identification and selection, as it is not feasible to obtain lung/immune cells in newborns. UCWBC provide a reasonable surrogate for our target organ/tissue (lung/immune cells) because they contain stem cells that can populate the lungs in later life and also provide a rich source of T cells [18] which are important producers of cytokines and other asthma mediators. The matched FPT provides ample tissue for obtaining fetal RNA to verify correlative changes in gene expression. A larger sample of 56 cohort subjects was used to validate the markers identified. Methylation of a 5′-CpG island (CGI) in *acyl-CoA synthetase long-chain family member 3* (*ACSL3*) was found to be positively and significantly associated with the level of maternal PAH exposure and with a parental report of asthma symptoms prior to age 5 in this exploratory study.

## Results

### Identification of candidate genes and selecting the most relevant candidate

**Figure 1:**
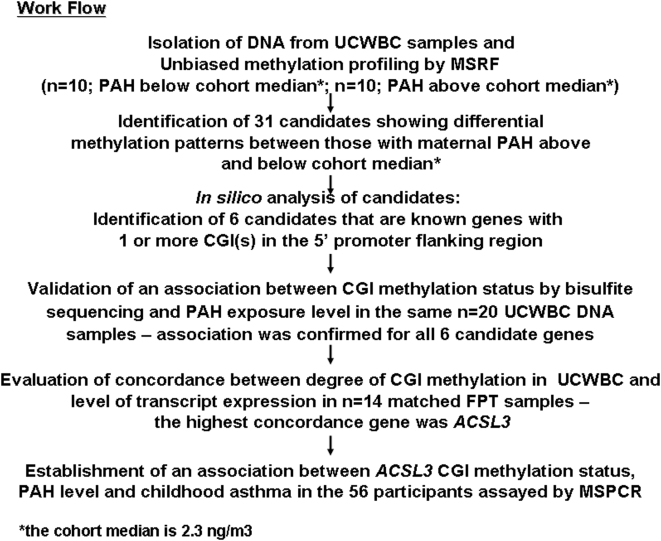
The workflow chart outlines how to discover the methylated genes in a step-by-step manner. Initially, ten umbilical cord white blood cell (UCWBC) samples from children with maternal (prenatal) PAH exposure above and ten with prenatal exposure below the cohort median were selected irrespective of gender and ethnicity. DNA was isolated from the sample was dichotomized according to the median of prenatal PAH exposure obtained from 48-hour personal air monitoring of the mothers during pregnancy (the cohort median is 2.3 ng/m3). Candidates with differentially methylation status between the samples from the two groups were discovered by Methylation Sensitive Restriction Fingerprinting (MSRF) following by subcloning and sequencing. Candidates were further identified by *in silico* analysis from databases from NCBI and UCSF genomic centre. Candidates which contained CpG island (CGI; CG rich region with >60% GC content and obs/exp ratio >0.6) on its 5′ flanking region were chosen for bisulfite sequencing to confirm the dependency of the methylation status of the CGI in a candidate gene on maternal PAH exposure. Six genes confirmed to have the latter relation were subjected to gene expression analysis in the 14 matched fetal placental tissues (FPT). The *ACSL3* was found to have the highest concordance between degree of CGI methylation and level of gene expression in reverse manner. It was therefore selected for the final analyses for correlation between methylation status of its CGI and transplacental PAH exposure and/or parent reported asthma symptoms of childhood asthma up to age 5 in a case-control study comprised of 56 participants. For this part of the study, Methylation specific-PCR (MSPCR) protocol was optimized to analyze the methylation status of R1 in Figure 4A was used as the high throughput method to analyze this larger sample set.

Figure 1 shows the work flow used in the discovery of candidates and strategies employed for selecting candidate genes associated with prenatal airborne PAH exposure and with asthma.

**Figure 2:**
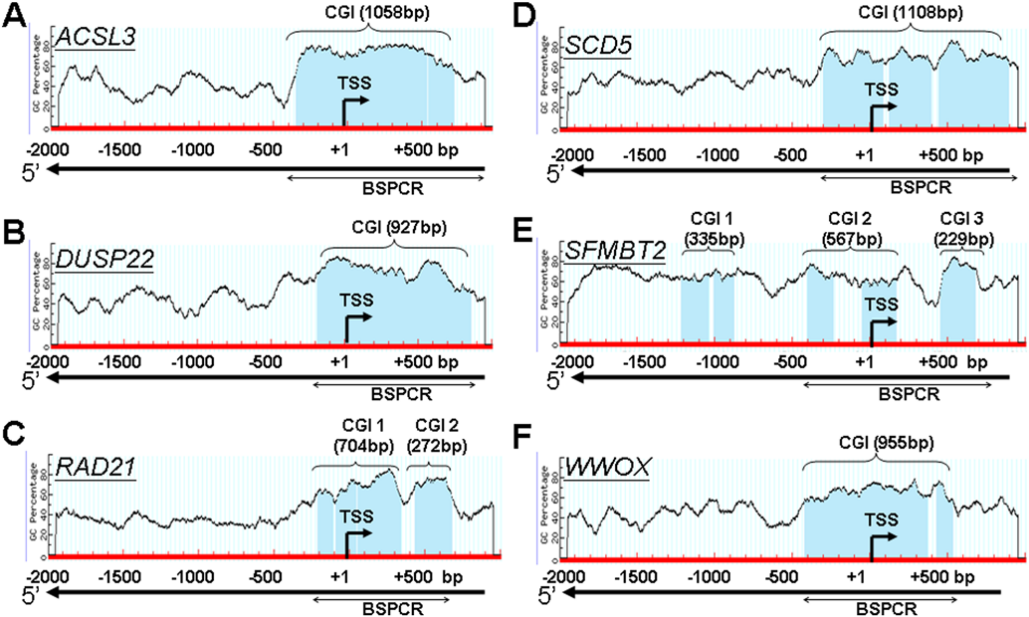
Schematic diagram of CG content (%) in the 5′ flanking region of A) *ACSL3*; B) *DUSP22*; C) *RAD21*; D) *SCD5*; E) *SFMBT2* and F) *WWOX*. The CGI(s) (shaded in blue in the genomic DNA sequence) were identified *in silico* based on a CG content >60%, with an observed/expected ratio of 0.6 according to instructions from MethPrimer. TSS: Transcription start site. The nested PCR-amplified region was indicated by a line with double arrow heads and methylation status of this region was determined by bisulfite genomic sequencing.

Twenty participants were randomly selected without knowledge of asthma classification: ten with maternal PAH exposure levels below the CCCEH cohort median of 2.3 ng/m^3^ (“low PAH group”) and ten with prenatal PAH levels above the cohort median (“high PAH group”). Methylation-sensitive restriction fingerprinting [19], [20] was performed to identify candidate sequences displaying differential methylation status between high and low PAH groups. Both hypo- and hyper-methylated candidate sequences in the high PAH group when compared to the low PAH group were selected. *In silico* analyses revealed that 19 out of 31 sequenced candidates were homologous to known genes (Table 1) and 6 of these aligned to a CGI(s) located in the 5′ flanking region (proximal promoter and/or exon/intron 1) of a known gene (5′-CGI) (Figure 2). These 6 genes therefore were chosen as candidates for further investigation. Unexpectedly, all six candidates were found be hypermethylated sequences in the high PAH group when compared to the low PAH group. A literature search showed that they are expressed in the lung and/or lymphoid tissues and have functions related to inflammatory/or other immune responses (*dual specificity phosphatase 22; DUSP22*, accession # BC022847, [21]), DNA damage and repair (*RAD21 homolog (S. pombe); RAD21*, accession # BC050381, [22]), fatty acid metabolism (*ACSL3*, accession # BC041692, [23]; *stearoyl-CoA desaturase 5; SCD5*, accession # NM_001037582, [24] ), gene transcription (*Scm-like with four mbt domains 2; SFMBT2*, accession # NM_001029880, [25]; *WW domain containing oxidoreductase; WWOX*, accession # AF211943, [26] ). Bisulfite sequencing analyses of the initial 20 UCWBC samples confirmed a good agreement between hypermethylation of all of the candidates' 5′-CGI(s) and either high or low PAH exposure status, consistent with MSRF findings (Table 1).

**Table 1:** Differentially methylated candidate genes with 5′CpG Islands identified with MSRF.

Clone	Pr1	Pr2	Hyper-methylation	Chr Band	Gene homology	Location	CGI at 5′ end	Gene Ontology
A1	20	21	Low PAH	8q24.3	NIK and IKKbetta-binding protein	INTRON15		NF-kappaB-inducing kinase acitivity
A2	20	21	High PAH	6p25.1	cDNA clone IMAGE:4825327	5′ END		
A3	20	21	High PAH	1q41	N/A			
A4	20	21	High PAH	19p13.3	cDNA clone FLJ14311	INTRON2		
A5	20	21	High PAH	10q26.3	N/A			
A6	20	21	High PAH	11q12.1	N/A			
**A7**	**20**	**21**	**High PAH**	**8q24.11**	**Protein in DNA double-strand break repair, RAD21**	**5′ END**	**<1 kb**	**Double strand break repair**
**A8**	**20**	**21**	**High PAH**	**6p25.3**	**Dual specificity phosphatase 22, DUSP22**	**INTRON1**	**<1 kb**	**Stress kinase involved in MAPK pathway**
A9	20	21	High PAH	8p23.1	Beta-defensin 107, DEFB107	3′ END		Defense response
B1	22	23	High PAH	1q32.2	mRNA for KIAA0463 protein, partial cds	INTRON4		
B2	22	23	High PAH	2p24.2	N/A			
B3	22	23	High PAH	3q24	WIAF-4002-STS Human THudson EST STS cDNA	INTRON1		
B4	22	23	High PAH	1q41	N/A			
D1	22	23	Low PAH	2p24.2	N/A			
D2	22	23	Low PAH	1q32.1	N/A			
D4	22	23	Low PAH	15q22.2	Vacuolar protein sorting-associated protein 13C, VPS13C	EXON7		Trans-Golgi to endosome transport
D5	22	23	Low PAH	1q41	N/A			
D6	22	23	High PAH	19p13.2	N/A			
**D7**	**22**	**23**	**High PAH**	**2q36.1**	**Acyl-CoA synthetase long-chain family member 3, ACSL3**	**INTRON1**	**<1 kb**	**Fatty acid metabolism**
D8	22	23	Low PAH	11q25	cDNA clone DKFZp686H1949	INTRON1		
D9	22	23	Low PAH	1p32.3	Clone BNGH42007037, similar to zinc finger protein GLI1	INTRON1		
D10	22	23	Low PAH	14q21.1	CTAGE-5B protein mRNA, complete cds, alternatively spliced	INTRON1		
**E1**	**7**	**11**	**High PAH**	**4q21.22**	**Stearoyl-CoA desaturase 5, SCD5**	**INTRON1**	**<1 kb**	**Fatty acid metabolism**
E2	7	11	High PAH	16p13.11	N/A			
E3	7	11	High PAH	16q24.2	Junctophilin 3, JPH3	INTRON3		Integral to membrane
E4	7	11	High PAH	Xq13.1	Ribosomal protein S4, X-linked	INTRON8		Translation
F1	10	11	High PAH	11q23.1	N/A			
**F3**	**10**	**11**	**High PAH**	**10p14**	**Scm-like with four mbt domains 2, SFMBT2**	**INTRON1**	**<2 kb**	**Gene transcription**
F4	10	11	High PAH	16q13	Small inducible cytokine A17 precursor, CCL17	INTRON2		Chemokine activity
G1	22	11	Low PAH	13q32.3	N/A			
**G2**	**22**	**11**	**Low PAH**	**16q23.1**	**WW domain-containing oxidoreductase isoform , WWOX**	**INTRON1**	**<1 kb**	**Gene transcription**

Note: Sequences were identified based on BLAT (UCSC genome center database) and RefSeq (NCBI database) search.

Abbreviation: NA, not available.

We next determined the strength of the relationship between the percent of methylation of a candidate gene's 5′-CGI in UCWBC DNA samples and its transcript expression level in matched FPT RNA samples (Table 2 and 3). The degree of methylation of a region encompassing the entire predicted 5′-CGI for each candidate gene was determined by bisulfite sequencing (Figure 2, the location of the amplicon was marked as BSPCR) and mRNA levels determined by real time RT-PCR. The percentage of promoter methylation (Met%) determined by bisulfite sequencing and relative gene expression levels (RER) determined by real time RT-PCR of all six candidates tested on 14 paired UCWBC DNA and FPT RNA samples are presented in Table 2. The degree of concordance between these two parameters for each candidate is shown in Table 3. We found that the degree of *ACSL3* 5′-CGI methylation and the level *ACSL3* transcript expression exhibited the expected inverse relationship with the highest concordance and significance (*τ* = −0.45; p<0.01) when compared to the other candidates. These findings indicate that the *ACSL3* 5′-CGI is likely a regulatory CGI since increased cytosine methylation in this sequence in UCWBC DNA samples was found to be significantly associated with transcriptional silencing of the gene in matched FPTs.

**Table 2:** Percentage of promoter methylation (Met%) in UCWBC DNA samples and relative expression ratio (RER) in FPT RNA samples of all six candiate genes*.

Sample		ACSL3		RAD 21		DUSP22		SCD5		SFMBT2		WWOX	
Low PAH	PAH level	Overall Met%	RER	Overall Met%	RER	Overall Met%	RER	Overall Met%	RER	Overall Met%	RER	Overall Met%	RER
#813	1.71	42	0.25	46	2.22	52	26.72	25	0.46	44	204.08	85	0.87
#826	1.80	45	0.48	56	0.58	42	9.31	33	0.71	36	3.06	85	0.74
#926	1.50	48	3.07	35	6.36	35	6984.8	33	1.17	68	1438.5	86	0.25
#954	1.15	35	0.26	36	0.66	36	12.70	33	0.00	55	79.09	78	0.04
#1066	1.22	67	0.43	45	0.79	36	2.85	60	57.70	36	9308.0	78	0.01
#1090	1.47	45	1.88	40	0.56	36	148.16	48	0.09	36	62.25	75	3.95
#755	0.85	45	0.03	35	1.19	44	13.45	33	1.41	44	173.87	85	0.13
**High PAH**	**PAH level**	**Overall Met%**	**RER**	**Overall Met%**	**RER**	**Overall Met%**	**RER**	**Overall Met%**	**RER**	**Overall Met%**	**RER**	**Overall Met%**	**RER**
#784	34.48	85	0.15	80	1.33	80	30.25	68	1.41	78	51.68	33	0.48
#831	2.41	81	0.34	90	2.18	85	5.15	68	0.95	68	0.29	51	0.16
#876	2.65	94	0.13	85	759.55	85	192.40	86	2.48	68	342.03	35	0.13
#901	2.82	95	0.14	85	0.80	85	10.72	85	0.19	75	15.07	35	0.97
#946	2.84	95	0.04	90	0.54	84	4.23	82	0.05	85	160.54	40	0.03
#1079	3.66	88	0.22	78	2.28	90	12.95	82	0.94	85	25.11	40	0.62
#1107	2.76	93	0.16	78	0.34	78	2.47	75	0.17	78	99.13	42	0.27

Note: ^*^Overall Met% is the percentage of methylated cytosine in the CGI(s) (regions mark as BSPCR in Fig 2) was determined by bisulfite sequencing; levels of gene expression normalized to β-actin level as a ratio using values of transcripts in FPT samples matched to UCWBC samples (n = 14) determined by real time RT-PCR.

**Table 3:** Concordance of degree of methylation of 5′CGI(s) in UCWBC samples and gene expression levels in FPT samples.

Gene Name	tau*	Confidence Interval	p-value
**ACSL3**	−0.45	[−0.76, −0.15]	<0.01
**RAD21**	−0.30	[−0.70, +0.09]	0.13
**DUSP22**	−0.31	[−0.66, +0.05]	0.09
**SCD5**	−0.11	[−0.49, +0.26]	0.55
**SFMBT2**	−0.23	[−0.57, +0.10]	0.18
**WWOX**	+0.22	[−0.11, +0.55]	0.20

Note: % of methylated cytosine in the CGI(s) (regions mark as BSPCR in Fig 2) was determined by bisulfite sequencing; levels of gene expression was measured as steady state transcript levels in FPT samples matched to UCWBC samples (n = 14) quantified by real time RT-PCR.

* The Kendall tau coefficient was calculated between % of methylation and gene expression; negative sign indicates inverse relationship. The larger the negative value means greater negative correlation and statistical significance was determined by a 2-tailed test with p<0.05.

**Figure 3:**
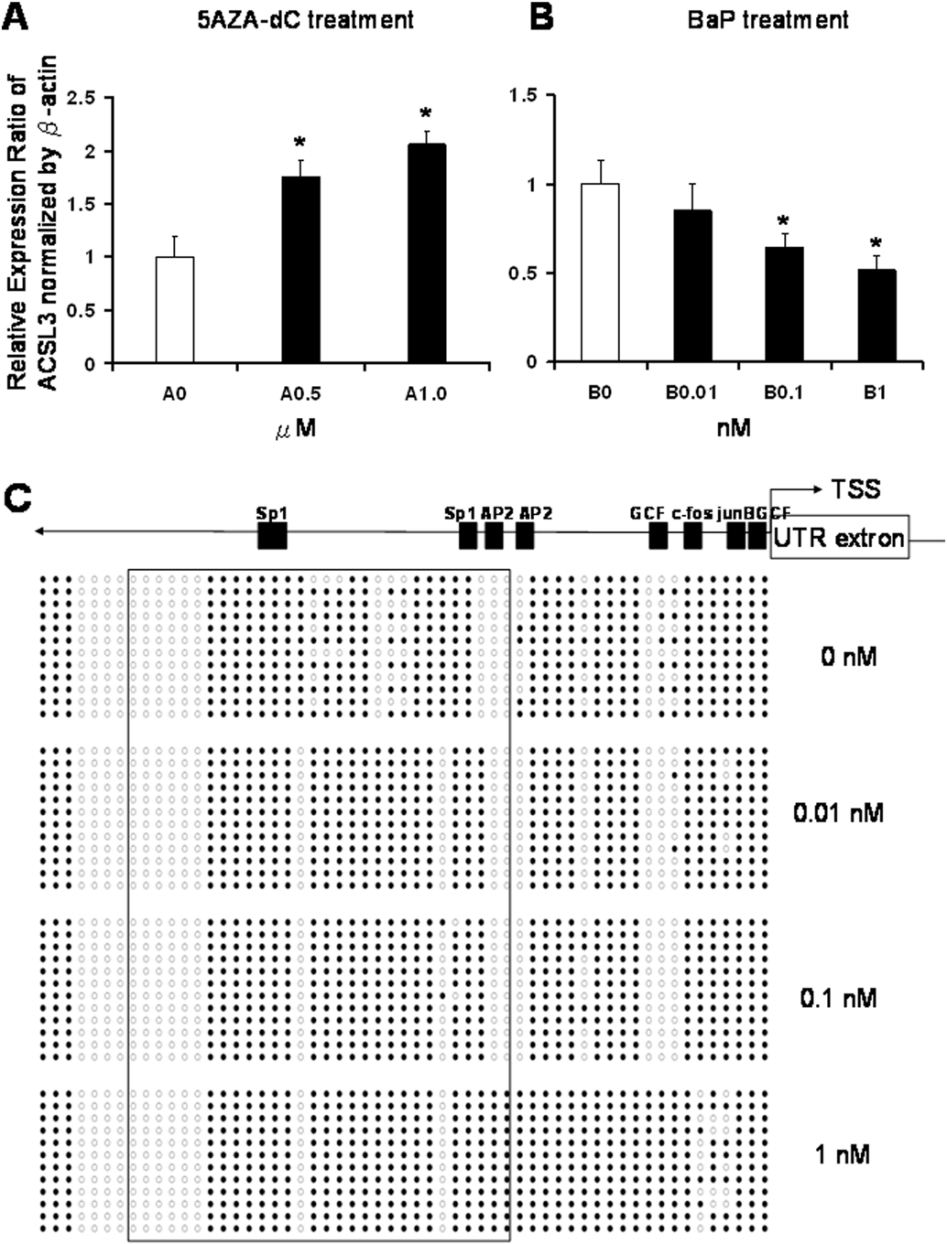
Real-time PCR analysis of gene expressions of *ACSL3* in non-small cell lung cancer H1299 cell line in response to (A) 5-aza-deoxycytidine (5AZA-dC) and (B) benzo[a]pyrene (BaP) and bisulfite genomic sequencing analysis of *ACSL3* promoter methylation status in response to BaP (C). A: Cells were treated with 0.5 and 1.0 µM 5-AZA-dC with DMSO as control every 2 days for a total of 8 days. B: Cells were treated with 0.01, 0.1 and 1.0 nM BaP with DMSO as control every 2 days for a total of 4 days. RNA was isolated, reverse transcribed and underwent real-time PCR. The 2-ΔΔCt method was used to calculate the relative expression level of transcripts normalized to *β-actin*. *Statistically significant differences between exposed and control was accepted at p<0.05. C: Diagram represents methylation status of *ACSL3* promoter of H1299 cells exposed to BaP assayed by bisulfite genomic sequencing. Cells were treated with 0.01, 0.1 and 1.0 nM BaP with DMSO as control every 2 days for a total of 4 days. Open circle: unmethylated CG; closed circle: methylated CG. Putative transcription factor binding sites such as Sp1, AP2, GCF, c-fos and junB are shown in scale with the particular CG sites on the promoter.

To further demonstrate that the methylation status of the *ACSL3* promoter affects gene expression, we used a lung non-small carcinoma cell line H1299 as a model system. Two series of experiments were performed. In the first series, we treated H1299 cells with a DNA demethylation agent, 5-aza-2′-deoxycytidine (5-aza-dC), and found that *ACSL3* expression was significantly increased (Figure 3A) along with promoter demethylation (data not shown). These data provide evidence that demethylation of the *ACSL3* promoter is associated with enhancement of gene expression. In the second series of experiments, H1299 cells exposed to low doses of benzo[a]pyrene (BaP; 0.01 to 1 nM), a representative PAH, showed a dose-dependent decrease in *ACSL3* transcript expression as indicated by real time RT-PCR analysis (Figure 3B) and increased promoter hypermethylation as revealed by bisulfite sequencing (Figure 3C). These data strongly support the notion that the CpG island in the ACSL3 promoter plays a regulatory role in gene expression.

### Association of methylation status of *ACSL3* 5′CGI with PAH exposure in the larger sample

*ACSL3* was chosen for subsequent assessment of the relationships between prenatal PAH exposure and gene 5′-CGI methylation status and for exploratory analyses of PAH exposure status and asthma classification in a larger sample of 56 children from the CCCEH cohort.

**Figure 4:**
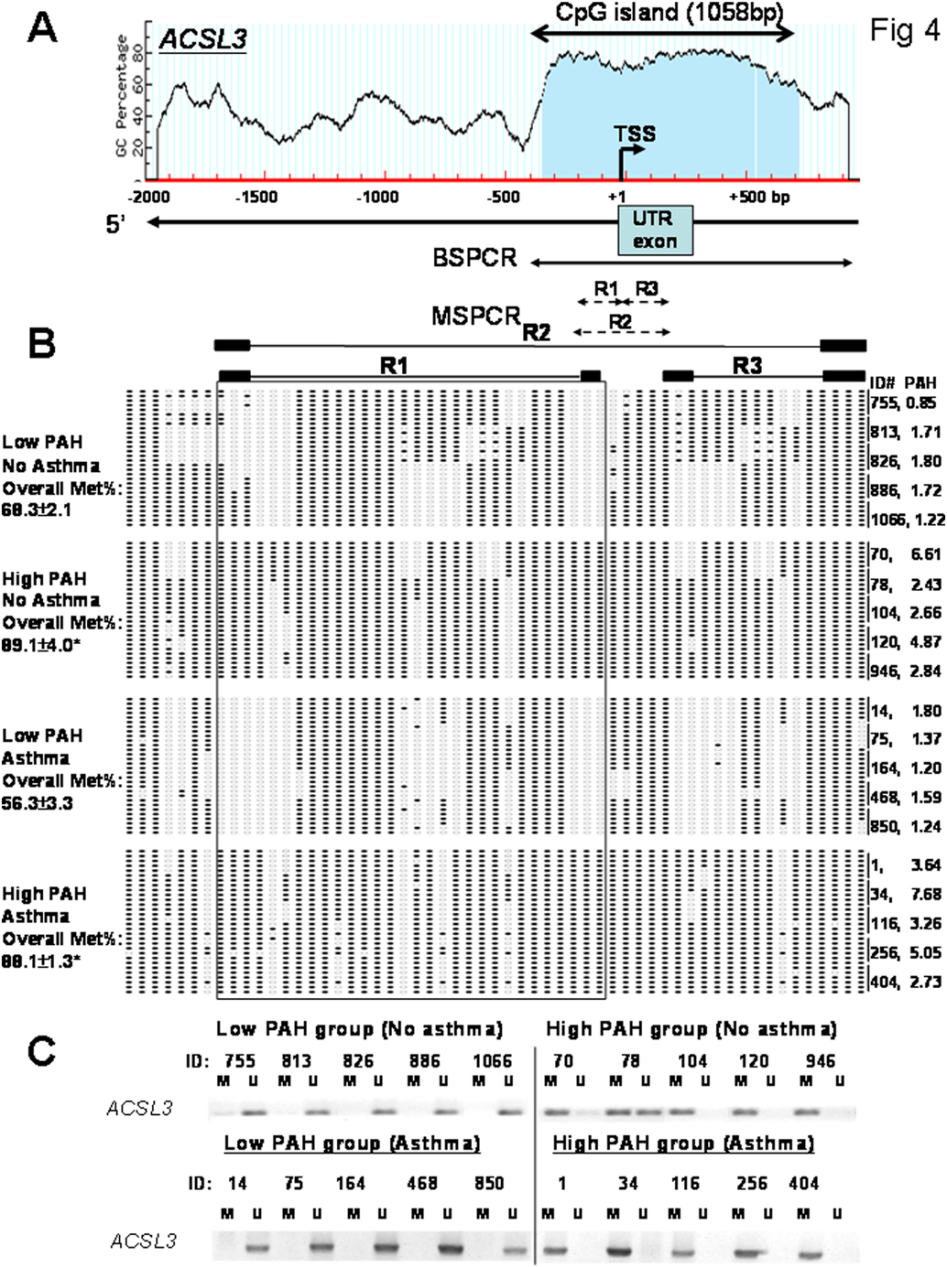
Methylation status of a region in the *ACSL3* 5′CGI analyzed by bisufite genomic sequencing and methylation specific PCR (MSPCR). A) Schematic diagram of the CG content (%) in the 5′ flanking region of the *ACSL3* gene. A CGI of 1058 bp was located at the 5′ end of *ACSL3* including transcription start site (TSS), untranslated region (UTR) exon1 and intron 1. Individual CG sites are marked as red vertical lines in the genomic DNA sequence. The PCR-amplified regions were indicated by lines and methylation status of this region were determined by BSPCR and MSPCR. B) The diagram represents the methylation status of MSPCR-amplified regions (R1–R3) of 20 samples with high or low PAH exposure with or without asthma assayed by bisulfite genomic sequencing. 6 clones were sequenced from each sample. The PAH level of each sample is shown. Open circle: unmethylated CG; closed circle: methylated CG. MSPCR-primers were then specially designed on particular regions (R1–R3). Overall Met% of *ACSL3* promoter is shown. Statistical difference was accepted at p<0.05* when compared with the group of low PAH without asthma. C) Representative results from MSPCR analyses on Region 1 (R1) of samples from the low and high PAH group. M: methylated; and U: unmethylated. The methylation status of *ACSL3* 5′-CGI of all samples was further analyzed by MS-PCR using the same sets of primers. Results are shown in Table 4 and Table 5. High PAH (≥ the cohort median of 2.3 ng/m3), Low PAH (<the cohort median).

The organization of the 5′-flanking sequence in the promoter region of *ACSL3* is shown in Figure 4A. Before we conducted the full investigation on the larger sample of 56 children, the following quality control experiments were performed. First, bisulfite sequencing analyses of an amplicon encompassing the entire predicted *ACSL3* 5′-CGI (Figure 4A, BSPCR marks the amplicon) were performed on the 20 UWBC DNA samples; five from each of the high or low PAH groups with or without asthma. Bisulfite sequencing data (Figure 4B) showed that hypermethylation of this CGI was found to be strongly associated with maternal exposure to a PAH level higher than the cohort median of 2.3 ng/m^3^. Asthma status did not seem to affect methylation status of *ACSL3* promoter. Second, based on bisulfite sequencing data, we designed and optimized three methylation-sensitive-PCR (MSPCR) protocols [27], [28] to amplify three regions (R1, R2 and R3) within the CGI that exhibited the greatest differential methylation changes between samples from the “high PAH” and the “low PAH” groups regardless of asthma status (Figure 4A, R1–3 are marked). These three regions were closely flanking the transcriptional start site (TSS). We further validated that the methylation status of R1 exhibited the strongest correlation with bisulfite sequencing data from analyses of the entire CGI. Representative data from MSPCR analyses of samples from children with prenatal PAH exposure above (high PAH) or below (low PAH) the cohort median are illustrated in Figure 4C. Thus, MSPCR of R1 was chosen as the high throughput method for investigating the methylation status of the *ACSL3* 5′-CGI in the larger sample comprised of 56 children.

**Figure 5:**
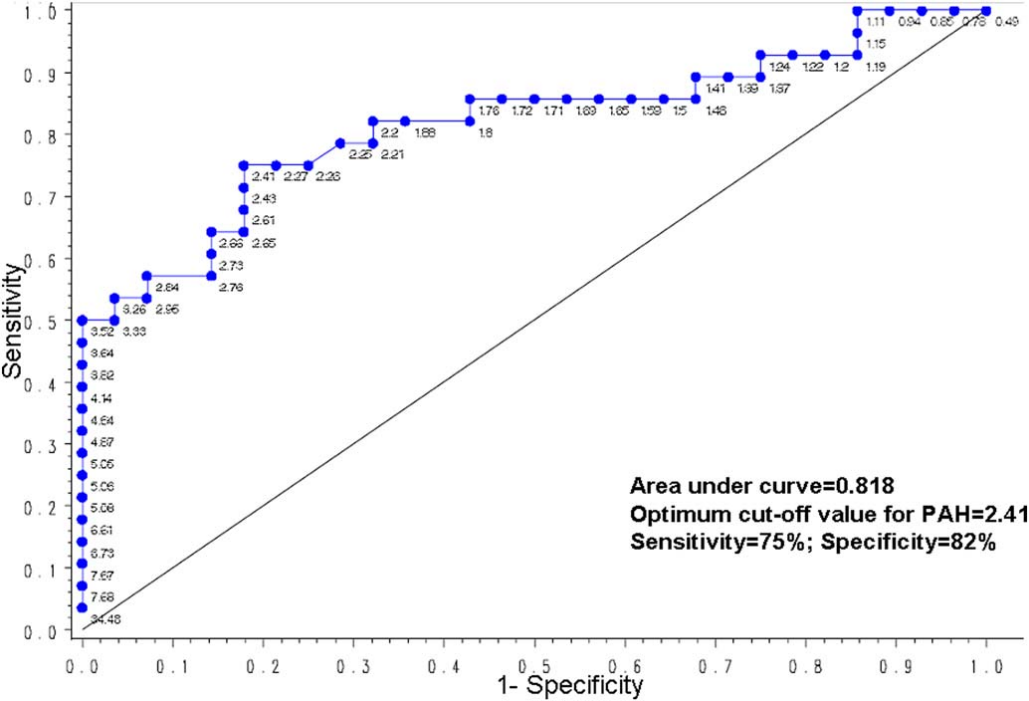
Receiver operating characteristics (ROC) plot for 56 children on PAH values and methylation status of *ACSL3* analyzed by MSPCR. Point labels are values of PAH of each children. Statistical software, sas, was used for ROC plot and R was used for determination of cutoff value. The cutoff value corresponds to the desired sensitivity and specificity (or 1-specificity).

Receiver Operating Curve (ROC) analysis of data from 56 subjects identified the maternal PAH exposure cutpoint value that best distinguished methylated versus unmethylated *ACSL3* 5′CGI status determined by MSPCR to be 2.41 ng/m^3^ (Figure 5). The Area Under ROC curve (AUC) value was 0.82 suggesting excellent separability of the subjects with methylated or unmethylated *ACSL3* 5′CGI. Using the “optimal” cutpoint value, methylation status was predicted with 75% sensitivity, 82% specificity.

Table 4 shows that 30 of the 56 children had a methylated *ACSL3* 5′-CGI; 81% of these 30 children were born to mothers with PAH exposure equal or exceeding 2.41 ng/m^3^, the cutpoint value determined by the ROC analysis. Thus, methylation was positively and significantly associated with maternal PAH exposure level. The odds ratio (OR) of having a methylated *ACSL3* 5′CGI in UCWBC DNA was 13.8, 95% CI = [3.8, 50.2] (p<0.001), given the maternal PAH exposure was ≥2.41 ng/m^3^, compared to those exposed to <2.41 ng/m^3^. *ACSL3* 5′CGI methylation status was not affected by gender or ethnicity.

**Table 4:** Number (%) of African Americans, Males, and Methylated Subjects by PAH Exposure Categories (<2.41, > = 2.41)^a^.

Characteristics*	PAH<2.41 (N = 30)	PAH≥2.41 (N = 26)	Subjects (N = 56)	Odds Ratios (95% CI's)
**Ethnicity (%AA)**	15 (50%)	15 (58%)	30 (54%)	1.4 (0.5, 3.9)
**Gender (% Males)**	12 (40%)	12 (46%)	24 (43%)	1.3 (0.4, 3.7)
**ACSL3 5′CGI Status (% Methylated)**	7 (23%)	21 (81%)	28 (50%)	13.8 (3.8, 50.2)^b^

Note: ^*^Odds Ratios, 95% Confidence Intervals (OR, 95%CI) measuring the odds of high PAH exposure in African Amercians (Compared to Dominicans), males (Compared to Females), and subjects with methylated ACSL3 5′CGI (compared to those with unmehtylated CGI) assayed by MSPCR (N = 56).

a The PAH cutpoint value 2.41 was obtained from an ROC curve where we considered that methylation status could be determined by PAH exposure level. We considered ACSL3 5′CGI to be methylated (determined by MSPCR) above 2.41 and unmethylated below 2.41. The value of the cutpoint determined the sensitivity (i.e. number of correctly classified unmethylated subjects or true positives), the specificity (i.e. number of correctly classified unmethylated subjects or true negatives), and the number of false positives and false negatives. The median of samples with PAH exposure <2.41 (N = 30) was 3.58 where that of samples with PAH exposure > = 2.41 was 1.55.

b p<0.001. Odds Ratio(OR) = 13.8, which may also be interpreted as the odds of ACSL3 5′CGI methylated when PAH = or >2.41 (compared to <2.41).

### Relationship between *ACSL3* 5′ CGI methylation and asthma classification

Table 5 shows the percentage of subjects with methylated or unmethylated *ACSL3* 5′ CGI according to asthma classification. Methylation of the *ACSL3* 5′CGI was positively and significantly associated with asthma classification. The odds ratio (OR) relating methylation status and asthma classification among all 56 subjects was 3.9, 95% CI = [1.1, 14.3] (p = 0.03). Eleven of 15 children classified as asthmatic (73%) had UCWBC DNA with a methylated *ACSL3* 5′-CGI. Table 5 further shows that the median (minimum, maximum) values of maternal PAH exposure differed significantly by methylation status of the *ACSL3* 5′-CGI: methylation = 3.39 ng/m^3^ (1.11, 34.5) and unmethylation = 1.70 ng/m^3^ (0.49, 3.33), respectively (p<0.001). All maternal PAH exposure values except the maximum (34.5 ng/m^3^) were below 7.7 ng/m^3^. When the latter analysis was repeated excluding the extreme high PAH value, the p-value was unchanged.

**Table 5:** Characteristics of Subjects (Number, % Column Total) by ACSL3 5′CGI methylation Status and Asthma Status Assayed by MSPCR (N = 56).

ACSL3 5′CGI Methylation Status	Methylated	Unmethylated	All	Odds Ratios (95% CI's)
Asthma (% Yes with asthma)^a^	11/28 (39%)	4/28 (14%)	15/56 (27%)	3.9 (1.1, 14.3)
Median PAH exposure with in each group^b^ (Min, Max)	3.39 ng/m^3^ (1.11, 34.48)	1.7 ng/m^3^ (0.49, 3.33)	2.26 ng/m^3^ (0.49, 34.48)	

Note: ^a^p = 0.03. OR = 3.9 measuring the odds of asthma given ACSL3 5′-CGI was methylated (M) verus unmethylated (U).

b p<0.001 testing the equality of median PAH exposure levels between subjects with methylated and unmethylated ACSL3 5′CGI in their UCWBCs.

## Discussion

In this exploratory study, we have identified *ACSL3* as a candidate biomarker/gene whose 5′-CGI methylation status appears to be related to transplacental PAH exposure and further associated with PAH-associated asthma. Thus, *ACSL3* may be the first potential surrogate endpoint for environmentally related childhood asthma. If confirmed, such a biomarker holds promise in assessing PAH exposure and as a clinically relevant predictor for asthma risk in children born to mothers exposed to air pollutants such as traffic-related combustion emissions.

We used a set of stringent criteria for the identification, selection and validation of candidates, which resulted in choosing *ACSL3* the final candidate for the 56-sample study. We first used an unbiased method, MSRF, to identify both hypo- and hyper-methylated sequences in the high PAH group compared to the low PAH group. *In silico* analyses then revealed that 19 out of 31 sequences identified were homologous to known genes and 6 of these aligned to a CGI(s) located in the 5′ flanking region of a known gene (5′-CGI). These 6 genes therefore were chosen as putative candidates for further investigation. By comparing the degree of methylation of their 5′CGI in UCWBC DNA samples and levels of gene expression in FPT RNA samples we identified *ACSL3* to have the highest inverse concordance *in vivo*. We then employed a lung cancer cell line (H1299) to demonstrate that the demethylation of *ACSL3* 5′-CGI by 5-aza-dC treatment induced enhancement of gene expression in H1299. Conversely, the treatment of H1299 with low doses of BaP, a PAH, was associated with dose-dependent hypermethylaiton of *ACSL3* 5-CGI and gene silencing. Collectively, these data strongly support methylation of the 5′-CGI of *ACSL3* in gene regulation. They also provide evidence that PAH could directly induce *ACSL3* promoter methylation. This well though out, step-wise approach allowed us to identify a candidate to be tested in the 56-sample study to determine whether methylation of the *ACSL3* 5′CGI could be a stand alone biomarker for PAH exposure and/or PAH-associated childhood asthma.

Measuring human exposure to chemicals such as PAHs and determining their relationship to disease outcomes is a challenge for environmental health, especially when children are the study subjects. Although some studies have found a positive correlation between PAH-induced DNA adducts and mutagenesis or cancer development [29] others have failed to establish clear cause-effect relations between PAH adducts and the induction of these or other disease outcomes [30], suggesting that other mechanisms may be involved. Moreover, some of the methods for measuring genotoxic mechanisms such as adducts are complex and low-throughput. Therefore, it is of significance that, in this investigative study, we have developed a relatively simple, high throughput assay based on methylation of a sequence in the promoter of the *ACSL3* gene that appears to distinguish children born to mothers with high airborne PAH exposure from those who were not (OR = 13.8; *p*<0.001) with high sensitivity (75%) and specificity (85%). The Area Under ROC curve (AUC) value for this assay was 0.82 suggesting excellent separability of the subjects with the methylated or unmethylated *ACSL3*sequence. This optimized MSPCR protocol allows for a rapid determination of *ACSL3* 5′-CGI methylation status in large sample sets, which is a normal requirement in population studies. It also has the added advantage to be developed into a minimally invasive test since only a small amount of DNA from UCWBC is required for the assay. If validated, such an assay is expected to have a high utility in the assessment of PAH exposure in future population-based studies.

Multiple epidemiological studies have suggested that asthma risk is determined by prenatal and/or early life exposure to pollutants and allergens that modify later life lung function and T helper cell allergic phenotype [11]. Epigenetic modifications of regulatory genes crucial to the development of asthma-related pathophysiology have been put forth as a possible mechanistic basis of this disease [11]. Thus, asthma may be caused by epigenetic changes such as DNA methylation, post-translational modifications of histones, dysregulation of non-coding RNAs, and chromatin alterations. At present, the best studied mechanism is DNA methylation-mediated disruption of gene regulation. In this regard, experimental data indicating that DNA methylation of genes critical to the differentiation of T helper cells could skew their response towards or away from an allergic phenotype lend support to this theory [31]–[34]. Additionally, we recently demonstrated that inhaled diesel exposure (a source of PAHs) and intranasal *Aspergillus fumigatus* induced specific DNA methylation changes in the promoters of *interferon-γ* and *interleukin-4* in T helper cells, respectively, and altered circulating levels of IgE in mice [35]. Despite progress made in experimental studies, fundamental questions related to asthma risk among human populations remain; these can only be addressed by cohort studies using specimens linked to well-annotated clinical and environmental exposure data. Such cohort-driven epigenetic research has the potential to address key questions, such as those concerning the influence of early life factors, environmental exposure, other disease states, and lifestyle factors on susceptibility to asthma. In this proof-of-principle study, we developed an innovative approach to population-based studies of environmental epigenetics, which could easily be implemented in future large-scale investigations.

A range of approaches is available to obtain quantitative and qualitative information on genomic DNA methylation changes [27]. We chose methylation sensitive restriction fingerprinting ( MSRF; [19], [20] ) as a tool for the discovery of PAH-reprogrammable genes in UCWBC due to its high sensitivity, requirement for very small amounts of starting materials (∼100 ng), and the ease in visual identification of differentially methylated sequences among a moderately large number of samples. Using only four arbitrary primers and six permutations of MSRF analyses on UCWBC samples from the high and low PAH exposure groups, we discovered 31 differentially methylated sequences. In future studies more arbitrary primers could be used to cover a larger portion of the genome in order to uncover more candidates [27]. In this study we had overcome another technical hurdle related to the quantification of gene expression in UCWBC. This task is challenging because the amount of cellular components in umbilical cord blood is usually very limited and prohibitive for high quality RNA isolation. To circumvent this problem, in a subset of samples, we assessed the concordance between the extents of methylation in the promoter-CGI(s) of each gene (using UCWBC DNA samples) with its respective transcript expression levels in RNA samples isolated from matched FPT samples. This choice was based on the logic that both UCWBC and FPT are fetal tissues are subjected to similar environmental exposure through the mother. Our results demonstrated good concordance for at least the *ACSL3* gene. In future design of such an epigenetic epidemiology experiment, preference should be given to improved techniques for the isolation of RNA and DNA from the UCWBC.

*ACSL3* belongs to the acyl-CoA synthetase long chain (ACSL) family of genes which encodes key enzymes in fatty acid metabolism [36]. *ACSL3* is expressed in lung and thymic tissue [37], [38]. Intracellular conversion of long chain fatty acids to acyl-CoA is required for energy production. ACSL catalyzes the initial step in this conversion yielding acyl-CoAs, which are used both in the synthesis of cellular lipids and in degradation via beta-oxidation for energy production [39], [40]. Moreover, acyl-CoA synthetase can catalyze the ATP-dependent production of arachidonoyl-CoA that is a substrate for lysophosphatidyl acyltransferase involved in arachidonic acid-containing phospholipid remodeling in proliferating T cells [41]. Lipid metabolism influences membrane proteins, including ion channels. In the lung, *ACSL3* is found most abundantly in microsomal and cell membrane fractions, with mitochondria as the next richest source [42]. Inhibition of ACS enzyme activity has been shown to cause cell death in *p53*-defective lung cancer cells [43]. Thus hypermethylation of this gene in T helper cells or lung tissues is expected to diminish fatty acid utilization and beta-oxidation-energy production, and possibly influence membrane phospholipid composition. Whether these functional changes directly affect asthma is unknown. However, several epidemiological studies show that fatty acid composition in milk [44], [45], diet [46], [47], and umbilical cord blood [48] affects the development of allergy and other inflammatory diseases including asthma. For example, dietary omega-3 (n-3) fatty acids having a variety of anti-inflammatory and immune-modulating effects may affect allergy and asthma. Further mechanistic studies in *in vitro* and *in vivo* experimental models are needed to decipher the role of *ACSL3* in childhood asthma. Interestingly, *ACSL3* is located in 2q36.1 which has recently been shown to be associated with regions of the asthma susceptibility loci, in specific populations [49]–[50]

Finally, the current finding of a putative epigenetic marker that is associated with PAH exposure and asthma adds to other evidence from the CCCEH cohort that PAHs increase risk of respiratory symptoms and probable asthma. At age 2 years, significantly more difficulty breathing and more probable asthma were reported among children jointly exposed prenatally to PAHs and postnatally to environmental tobacco smoke (ETS) (OR for probable asthma = 7.52, 95% CI 1.71–33.11, p<0.01) [3]. These relationships were reexamined through age 5 years and similar trends were detected, with an observed interaction between high prenatal PAH exposure and postnatal ETS on wheeze and probable asthma at age 5 (OR = 5.48, 95% CI 1.17–25.58, p<0.01) (unpublished data). A parallel cohort study in Krakow, Poland has found a significant relationship between prenatal PAH exposure and respiratory symptoms [51]. In a subset of 333 subjects, PAH exposure measured during pregnancy by personal air monitors was associated with an increased risk for wheezing without cold (RR 3.8; 95% CI: 1.2–12.4), during the course of the infant's first year of life [51].

In conclusion, no previous studies have examined the effects of prenatal exposure to ambient air pollutants on DNA methylation patterns in genes potentially associated with the asthma phenotype of the offspring. This is an important gap in our understanding of asthma pathogenesis. Given the potential importance of epigenetic mechanisms in the etiology of childhood asthma, research is needed to identify an epigenetic profile related to PAH-associated childhood asthma and determine whether reprogramming events associated with transplacental exposure to PAHs increase risk of childhood asthma. The current report of *ACSL3* as a candidate biomarker of PAH exposure and a putative predictor of PAH-associated childhood asthma represents an important first step in this area of research. Finally, the novel design of this study could serve as a prototype approach for future discoveries of additional exposure biomarkers or surrogate disease endpoints in other birth cohort studies.

## Materials and Methods

### Study Population

Study subjects were nonsmoking Dominican and African American women and their children residing in Washington Heights, Central Harlem and the South Bronx participating in the CCCEH cohort study previously described [4]–[6]. Written informed consent was obtained from all subjects following procedures approved by the Institutional Review Board of the New-York Presbyterian Medical Center. Full enrollment into the study required completion of prenatal air monitoring and collection of a maternal and/or umbilical cord blood sample at delivery. As of November 1, 2007, 74% of the more than 600 fully enrolled subjects had been retained in the cohort through at least five years. During pregnancy and following delivery, a trained bilingual research team administered repeat questionnaires eliciting environmental and respiratory health histories [4]. There were no differences in demographics of subjects fully enrolled and those lost to follow-up [4]–[6]. Based on parental report of a doctor's diagnosis of asthma or probable asthma before the age of five, children were classified as “asthmatic” or “nonasthmatic.” (“with or without a parental report of asthma”).

### Study Sample Selection

All subjects with stored UCWBC and paired FPT were eligible for inclusion in this proof-of-principle study. The population was dichotomized on the median PAH value of the entire cohort (2.3 ng/m^3^). Half of the 56 subjects in the present study were selected from among the ones above this median (the high PAH exposure group) and half from among those below the cohort median (the lower PAH exposure group). Twenty subjects (10 high and 10 low exposure) were used to identify UCWBC DNA sequences whose methylation status was dependent on maternal PAH exposure (high versus low) and to validate concordant transcript changes in matched FPT samples. All 56 samples were then used to validate the association between PAH exposure and *ACSL3* 5′CGI methylation and to explore the association between *ACSL3* 5′CGI methylation and childhood asthma status. Table 6 shows the characteristics of the cohort population and study sample, respectively. The sample was representative of the cohort population in terms of maternal age, child's sex, median PAH exposure, and percentage of children with probable asthma; however, the sample differed with respect to ethnicity. Table 7 showed the PAH distribution and variation in PAH levels in selected and unselected cohorts.

**Table 6:** Population characteristics.

Category	Subcategory	Full cohort (N = 729)	Study sample (N = 56)	p-value
**Maternal ages (yrs)(mean±SD)**		25.1±4.9	25.4±4.6	0.65
**Baby's sex (%)**	**Male**	48.3	42.9	0.40
	**Female**	51.7	57.1	
**Ethnicity (%)**	**Dominican**	63.5*	46.4	<0.01
	**African American**	36.5	53.6	
**Median total PAH (ng/m^3^)**		2.3	2.3	0.83
**Probable asthmatic up to 5 years (%)**		27.2	26.8	0.95

Note: ^*^Among N = 606 samples with available data.

**Table 7:** Total maternal PAH exposure levels in the study sample as compared to the full cohort.

	N	Min	p25	p50	p75	Max	Geomean	95% CI
Included in study	56	0.49	1.48	2.26	3.43	34.48	2.37	1.96, 2.86
Not included*	546*	0.27	1.45	2.29	3.69	145.00	2.38	2.23, 2.53

Note: ^*^There are 127 participants in the full cohort for whom we are missing prenatal PAH. No significant differences by ttest (means) or kruskal-wallis (medians). p25, p50 and p75 denote means at the 25, 50 and 75 percentile.

### Monitoring and Sample Collection

Personal prenatal air monitoring was conducted for two consecutive days on all participants during the 3^rd^ trimester of pregnancy as previously described [4]–[6]. Levels of pyrene and eight carcinogenic PAHs were analyzed (4). Total PAH levels for each participating mother were computed as the sum of the eight carcinogenic PAHs, where the values below the limit of detection were imputed as 0.125 ng/m^3^ (the median value between 0 and the limit of detection). Umbilical cord blood (30–60 mL) was collected at delivery. The buffy coat, packed red blood cells, and plasma samples were separated and stored at −80°C. DNA (100 ng–500 ng) was extracted from UCWBCs. For some UCWBC samples, matched FPTs were also available and total RNA was extracted using a TRIZOL® (Invitrogen, CA) according to manufacturer's protocol. All samples were coded.

### Methylation Sensitive Restriction Fingerprinting (MSRF)

MSRF was performed in a single-blinded manner on asthma classification, as previously described [19], [20]. Five hundred ng of UCWBC DNA was restriction-digested using *Mse*I [New England Biolabs, MA] alone or in combination with *Bst*UI [New England Biolabs, MA] and amplified by PCR with various combinations of paired arbitrary primers (Table 8). Following electrophoresis, candidate sequences displaying differential methylation status between the “high PAH group” and “low PAH group” were isolated, extracted from the gel, cloned, identified and aligned with various databases. Sequences matched to known genes and with 5′-CGIs were selected for bisulfite genomic sequencing and confirmation studies of their regulatory function in gene expression. Six candidates [*ACSL3*, *RAD21*, *DUSP22*, *SFMBT2*, *SCD5 and WWOX*] were selected.

**Table 8:** Primers for Methylation Sensitive Restriction Fingerprinting (MSRF), Bisulfite Genomic Sequencing, Methylation Specific PCR (MSPCR) and Real-time PCR.

**Methylation Sensitive Restriction Fingerprinting**	
***Bs7***	5′- GAGGTGCGCG-3′
***Bs10***	5′- AGGGGACGCG-3′
***Bs11***	5′- GAGAGGCGCG-3′
***Bs20***	5′- GCGCCGACGT-3′
***Bs21***	5′- CGGGACGCGA-3′
***Bs22***	5′- CCGCGATCGC-3′
***Bs23***	5′- TGGCCGCCGA-3′
**Bisulfite Genomic Sequencing**	
***hBS_ACSL3_F1***	5′-GGATTTGATAGTAATTTTTGTAAGAA-3′
***hBS_ACSL3_R1***	5′-ACCTAATAACACCTACCCCACAAA-3′
***hBS_RAD21_F1***	5′-TTTAAAATAAGTATAAGAATAAAGTTAAA-3′
***hBS_RAD21_R1***	5′-AAAAAACAACAACAATCACTAACC-3′
***hBS_DUSP22_F1***	5′-GTTAAAGGGGGATTTGGAGATT-3′
***hBS_DUSP22_R1***	5′-TCACAAACACACACACAAAATCTAA-3′
***hBS_SCD5_F1***	5′-GGGATTGGAAAAATATTATAAAAGGTATT-3′
***hBS_SCD5_R1***	5′-ACAAATTCCCACTAACCACATAAAA-3′
***hBS_SFMBT2_F2***	5′-TTAAGATTGTTTTGGGGATTGAAT-3′
***hBS_SFMBT2_R2***	5′-TCCTAAACTAAAAAAAACCCCTAAC-3′
***hBS_WWOX_F1***	5′-TGTAAAATAAAGGTTGTGAATTAGTA-3′
***hBS_WWOX_R1***	5′-ACCCCTACACTCCAACCTAAATAAC-3′
**Methylation Specific PCR**	
***MS_ACSL3_MF-R1***	5′-TTTTTGGTCGATTTCGTTTTC-3′
***MS_ACSL3_MR-R1***	5′-AAACTACCACCCATTAACCCG-3′
***MS_ACSL3_UF-R1***	5′-GTTTTTTTTGGTTGATTTTGTTTTT-3′
***MS_ACSL3_UR-R1***	5′AAACTACCACCCATTAACCCAC–3′
***MS_ACSL3_MF-R2***	5′-TCGTTTTTTTTGGTCGATTTC-3′
***MS_ACSL3_MR-R2***	5′-ATACGAAAAAACGAACGTATACGAC-3′
***MS_ACSL3_UF-R2***	5-GTTTGTTTTTTTTGGTTGATTTTGT-3′
***MS_ACSL3_UR-R2***	5′-CAATACAAAAAAACAAACATATACAAC-3′
***MS_ACSL3_MF-R3***	5′-TTTAGGCGGTTTCGTTTAATAGAC-3′
***MS_ACSL3_MR-R3***	5′-ATACGAAAAAACGAACGTATACGAC-3′
***MS_ACSL3_UF-R3***	5′-TTAGGTGGTTTTGTTTAATAGATGT-3′
***MS_ACSL3_UR-R3***	5′-ATACAAAAAAACAAACATATACAAC-3′
**Real-time PCR**	
***hACSL3_F1***	5′-GAGAGTTTGAACCCGATGGA-3′
***hACSL3_R1***	5′-TTGGCACAACAAATCCAATG-3′
***hRAD21_F1***	5′-AGAGCTTCCCCCAGAAGAAC-3′
***hRAD21_R1***	5′-AAGAGCACGCTGAAGACCAT-3′
***hDUSP22_F1***	5′-AGGAGCGTGACACTGGTGAT-3′
***hDUSP22_R1***	5′-CTGCCGATACTGATGGACCT-3′
***hSCD5_F1***	5′-GACCTGCTTGCTGATCCTGT-3′
***hSCD5_R1***	5′-GGGCTGATGTGCTTGTCATA-3′
***hSFMBT2_F1***	5′-GGGATTGGGTAATTTTATTTTTTTT-3′
***hSFMBT2_R1***	5′-GACCTCCGTTTCTTCTGCAC-3′
***hWWOX_F1***	5′-CGGATGGGAACAAGAAACTG-3′
***hWWOX_R1***	5′-CAACCACTTTGCCAGTGAAA-3′

### Treatment of H1299 with 5-aza-dC and BaP

H1299 cell line is a gift from Dr. Carolyn M. Klinge (University of Louisville) and were maintained in RPMI-1640 medium (Invitrogen, CA) supplemented with 10% fetal bovine serum (Hyclone, UT), 10 mM HEPES (Invitrogen, CA), 1 mM sodium pyruvate (Invitrogen, CA) and 4500 mg/L glucose (Invitrogen, CA). For demethylation assays, cells were treated with 0.5 and 1.0 µM 5-aza-dC (Tocris, MO) with DMSO (Sigma, MO) as control every 2 days for a total of 8 days. For BaP treatment, cells were exposed to 0.01, 0.1 and 1.0 nM BaP (Sigma, MO) with DMSO as control every 2 days for a total of 4 days. Medium was replenished with new drug of 5-AZA-dC or BaP every 48 hr. DMSO was added in the control samples. RNA was isolated, reverse transcribed and undergone real-time PCR. DNA was isolated, bisulfite-modified and undergone bisulfite genomic sequencing.

### Bisulfite genomic sequencing

Genomic DNA (100 ng) from UCWBC samples or H1299 cells was modified with sodium bisulfite by using the EZ Methylation Modification Kit (Zymo Research, CA) before conducting PCRs. *In silico* analyses and detailed database searches were used to predict the 5′-CGI(s) in each gene. Primers were designed to amplify ∼0.9 to 1.4 kb fragments encompassing the 5′-CGI(s) of a candidate gene from bisulfite-modified DNA (Table 8). Amplicons were generated from individual UCWBC sample and subcloned into the pCR2.1 vector (Invitrogen, CA); eight or more clones were picked and sequenced (Macrogen, Korea).

### MSPCR analyses

Bisulfite-modified genomic DNA was subjected to MSPCR analyses [27], [28]. Three sets of primers were designed to amplify regions (Figure 3A; R1, R2, and R3) within the 5′-CGI that exhibited the highest degree of differential methylation between the high and low PAH group. For each region, the M primer set amplifies the target sequence when it is methylated; the U primer set only amplifies the unmethylated allele (Table 8). Thirty-five-cycle PCRs were performed with 1 unit Platinum Taq DNA polymerase (Invitrogen, CA) using the following conditions: denature at 94°C for 30 s, anneal at 58°C for 1-min, and extend at 72°C for 1-min, followed by 12-min final extension. PCR products were separated on 2% agarose gel and visualized with ethidium bromide.

### Real-time reverse-transcriptase-PCR (real time RT-PCR)

Total RNA was isolated from FPT samples matched to UCWBC samples or from H1299 cells as previously reported [14], [16]. Steady state mRNA levels of the genes under investigation were quantified by SYBR Green-based real time RT-PCR with normalization to *β-actin* as previously described [14], [16]. Primers specific for quantification of transcripts of the selected genes are listed in Table 8. Three separate real time RT-PCRs were performed on each FPT sample to obtain a mean value. The 2-ΔΔCt method was used to calculate the relative expression level of transcripts normalized to *β-actin*. The mean value from a universal reference RNA (Clontech, CA) was arbitrarily assigned an abundance value of 1.00 for each gene. Mean values from all other samples were normalized against this sample.

### Statistical methods

The Kendall coefficient of concordance (tau) was calculated to measure the strength of the relationship between the percent of methylation of a gene's promoter CGI in UCWBC DNA samples and its transcript expression level in matched FPT RNA samples. Ninety-five percent confidence intervals and 2-tailed p-values were obtained to determine the expected range and significance of observed tau values.

Chi-square tests were performed to investigate possible differences in the percents of African Americans, males, and methylated subjects between categories of PAH exposure, (<2.41, ≥2.41). Odds ratios and 95% confidence intervals of high PAH exposure for each of the three characteristics were calculated and tested for statistical significance. The cutpoint of 2.41 was determined from an ROC curve analysis following logistic regression of methylation status on continuously measured PAH exposure. This value separated methylated and unmethylated subjects according to ‘high/low’ PAH exposure. We considered MSPCR to be methylated above 2.41 and unmethylated below 2.41.

Logistic regression was performed to evaluate the association between parental report of asthma (dependent variable) and methylation status (independent variable). First, methylation status was modeled alone; then gender and ethnicity were added to the model. The parameter estimate and standard error of methylation status changed less then 15% when adjusted for gender and ethnicity, therefore the unadjusted odds ratio was presented. The equality of the median PAH exposure levels between methylated and unmethylated subjects was tested by the non-parametric Kruskal-Wallis test. For all analyses a 5% alpha level indicated significance unless stated otherwise. Analyses were performed using SAS, Version 9.1 (SAS Institute, Cary, NC).
