# Correction: Relation of DNA Methylation of 5′-CpG Island of *ACSL3* to Transplacental Exposure to Airborne Polycyclic Aromatic Hydrocarbons and Childhood Asthma

**DOI:** 10.1371/annotation/6a678269-9623-4a13-8b19-4e9431ff3cb6

**Published:** 2009-08-18

**Authors:** Frederica Perera, Wan-yee Tang, Julie Herbstman, Deliang Tang, Linda Levin, Rachel Miller, Shuk-mei Ho

Figure 3 is incorrect. A detailed explanation can be found in the Comments section of this article. Please view the corrected figure and legend here: 

**Figure 3:**
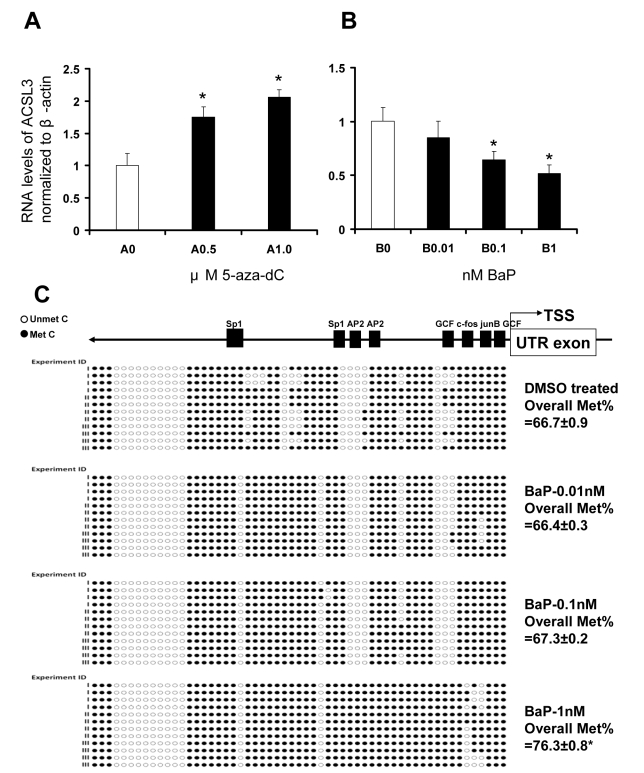
Real-time PCR analysis of RNA levels of *ACSL3* in H1299 cells in response to (A) 5-aza-deoxycytidine (5AZA-dC) and (B) benzo[a]pyrene (BaP). (C) Methylation status of the *ACSL3* promoter in response to BaP by bisulfite genomic sequencing. A: Cells were treated with 0.5 or 1.0 μM 5-AZA-dC or with DMSO (0.1%) as control every 2 days for 8 days. B: Cells were treated with 0.01, 0.1 or 1.0 nM BaP or with DMSO (0.1%) as control every 2 days for 4 days. RNAs were isolated, reverse transcribed and ACSL3 expression was quantitated by realtime PCR. The 2-ΔΔCt method was used to calculate the relative transcript level which normalized to *β-actin*. Data are presented as the mean ± standard deviation of three experiments. *p-values <0.05 were considered statistically significant (compared to control). C: Diagram represents methylation status of the *ACSL3* promoter of H1299 cells exposed to BaP determined by bisulfite genomic sequencing. Cells were treated with 0.01, 0.1 or 1.0 nM BaP or with DMSO (0.1%) as control every 2 days for 4 days. DNA was isolated and subjected to bisulfite genomic sequencing. Four individual clones from each experiment for each BaP concentration were sequenced and triplicate experiments were performed (shown with individual experiment ID (I-III). A total of 12 clones from each BaP concentration were sequenced. BiQAnalyzer was used to convert bisulfite-treated DNA sequences back to original genomic DNA sequences (including the reverse complement of each sequence). In order to avoid misalignment in the BiQAnalyzer, all flanking vector sequences were removed from original sequences prior to analysis. Sequence alignments were performed using CLUSTAL W (1.83). Methylated/Unmethylated CpG sites were visualized and exported to Microsoft Excel for methylation map generation. Each circle represents a CpG site within the *ACSL3* promoter. A total of 57 CpG sites were analyzed. Open circles represent unmethylated CpGs and closed circles represent methylated CpGs. Putative transcription factor binding sites such as Sp1, AP2, GCF, c-fos and junB are shown in scale on the promoter. Overall percentage of CpGs at the *ACSL3*promoter methylation is shown. The difference in percentage of CpG promoter methylation between 1nM BaP and control samples is statistically significant (p<0.01) as denoted by asterisk * next to the percentage.

